# Αlpha-Synuclein as a Mediator in the Interplay between Aging and Parkinson’s Disease

**DOI:** 10.3390/biom5042675

**Published:** 2015-10-16

**Authors:** Wojciech Bobela, Patrick Aebischer, Bernard Laurent Schneider

**Affiliations:** Brain Mind Institute, Ecole Polytechnique Fédérale de Lausanne (EPFL), Lausanne 1015, Switzerland; E-Mails: wojciech.bobela@epfl.ch (W.B.); patrick.aebischer@epfl.ch (P.A.)

**Keywords:** alpha-synuclein, Parkinson’s disease, aging, mitochondria, proteostasis, nigral dopaminergic neurons, metabolism

## Abstract

Accumulation and misfolding of the alpha-synuclein protein are core mechanisms in the pathogenesis of Parkinson’s disease. While the normal function of alpha-synuclein is mainly related to the control of vesicular neurotransmission, its pathogenic effects are linked to various cellular functions, which include mitochondrial activity, as well as proteasome and autophagic degradation of proteins. Remarkably, these functions are also affected when the renewal of macromolecules and organelles becomes impaired during the normal aging process. As aging is considered a major risk factor for Parkinson’s disease, it is critical to explore its molecular and cellular implications in the context of the alpha-synuclein pathology. Here, we discuss similarities and differences between normal brain aging and Parkinson’s disease, with a particular emphasis on the nigral dopaminergic neurons, which appear to be selectively vulnerable to the combined effects of alpha-synuclein and aging.

## 1. Introduction

The implication of alpha-synuclein (α-syn) in familial cases of Parkinson’s disease (PD) is the most compelling evidence for the critical role that this protein plays in pathogenesis. Missense mutations leading to changes in the amino acid sequence of α-syn [1,2,3,4,5], as well as multiplication of the α-syn gene [6,7], demonstrate that changes in the conformation and the abundance of this protein are important factors that can lead to disease. The dominant mode of disease inheritance [1,2,3,4,5], together with experimental findings, suggests that α-syn gains toxic functions leading to progressive neurodegeneration. Importantly, similar mechanisms are likely to contribute to the majority of PD cases (90%) that are considered as idiopathic [1,2,3]. Indeed, α-syn has been identified as the major component of Lewy Bodies (LBs) and Lewy Neurites (LNs), two forms of the protein aggregates that are characteristic of PD [8,9]. In addition, a prominent link between genetic variations in the α-syn gene locus and the occurrence of PD has been established [10,11,12].

While it is evident that α-syn is a key actor in PD pathogenesis, its exact role, although extensively investigated, is not yet fully understood, neither in healthy nor in diseased neurons [13,14]. Nevertheless, it is increasingly recognized that the toxicity of α-syn becomes fully apparent only when neuronal cells are exposed to additional stressors. Understanding the multiple risk factors that modulate the susceptibility to disease is therefore key. Some of these factors can be of environmental origin, such as exposure to mitochondrial toxins (e.g., rotenone, MPTP and paraquat) and heavy metals [15,16,17]. Of particular importance is brain aging, as PD prevalence clearly increases over time, to reach 1% at the age of 60 and 5% for people over 85 years old [18,19,20]. In addition, although the pattern of α-syn pathology covers many regions of the central nervous system (CNS), only specific neuronal populations appear to undergo neurodegeneration [20,21]. While the common understanding is that α-syn misfolding and deposition may propagate across specific neuronal networks [14,22], it is critical to explore why the dopamine (DA) neurons of the *substantia nigra pars compacta* (SNpc) are selectively vulnerable to the pathogenic changes [23].

Regarding aging as a biological process, we can define this phenomenon at the cellular level as a progressive inability to efficiently recycle cellular components such as organelles and macromolecules. In addition to the progressive accumulation of molecular damage inside the cells, aging is associated with an overall decrease in proteasome activity, impaired autophagy, and mitochondrial dysfunction [24,25,26,27,28,29]. Postmitotic neurons are expected to be more susceptible to the cumulative effects of aging as they do not self-renew by dividing. Here, we highlight similarities and differences between the normal aging and the pathologic process observed in PD. We discuss how the pathogenic effects of α-syn accumulation may interact with the consequences of aging on the human brain. In particular, we present current hypotheses for the selective vulnerability of nigral DA neurons in the context of PD.

## 2. The Physiological Role of α-syn

Alpha-syn, along with beta- and gamma-synucleins, are members of a family of small acidic proteins expressed only in vertebrates [30]. Synucleins are very abundantly present in the CNS [31]. The SNpc and hippocampus are the brain structures with the highest α-syn protein levels [9]. The fact that α-syn is expressed in such a significant quantity suggests that this protein might play an important role in neuronal homeostasis. Alpha-syn is a 140 amino acid protein, encoded by six exons, and which contains three distinctive domains: an *N*-terminal domain which adopts a alpha-helical structure upon binding to cellular membranes; a median domain called non-amyloid component (NAC) which is normally folded as an alpha helix, but is prone to acquire a beta-sheet conformation; and an acidic non-folded C-terminal domain [14]. Although α-syn can be located in the nuclear and synaptic compartments—hence the name “synuclein”—it is actually mostly confined to the cytosol and appears highly enriched in synapses. Alpha-syn has been shown to interact with lipid membranes, particularly those of synaptic vesicles [14,30]. In the cell nucleus, α-syn interacts with histones and controls the levels of its own expression [32].

### 2.1. Alpha-Synuclein and the Synapse

The exact role of α-syn is still not fully elucidated yet. Interestingly, α-syn null mice are not only viable, but also do not show any significant motor or cognitive impairment, except for some mild defects in synaptic transmission [33,34,35]. The reason for this mild phenotype is most probably related to compensatory mechanisms involving either beta- or gamma-synuclein. Indeed, although triple knockout mice for all synuclein genes are viable, they show within the SNpc a modest progressive decrease in the expression of tyrosine hydroxylase (TH), a rate-limiting enzyme in DA synthesis [31]. The most striking observation, however, is that these animals have hippocampal synapses that are 30% smaller than those found in their wild-type counterparts [31]. Furthermore, their neurons have increased axon diameters and lower levels of axonal myelination, which leads to slower propagation of the action potentials [31]. Since these animals display significantly smaller synapses, with drastic alterations in the synaptic protein composition, it has been proposed that one of the main roles of synucleins might be to promote the maturation and control the stability of established synapses [31]. The former point is supported by the observation that α-syn level peaks during the postnatal period [36,37,38].

Most of the well-established effects of α-syn rely on its propensity to bind lipid membranes on synaptic vesicles, mitochondria or Golgi apparatus. Alpha-syn serves a role of molecular chaperone, facilitating SNARE complex assembly at the synapse [39,40,41]. Furthermore, it controls the dynamics of neurotransmitter release and clathrin-dependent replenishment of the pool of synaptic vesicles [33,34,42], as well as potentially participates in the stabilization of mitochondrial membrane proteins [43].

Some results also point to the role of human α-syn in regulating DA neurotransmission. Transgenic mice have been generated that express human wild-type or mutated α-syn under the control of the α-syn gene regulatory sequences. A “knock in” mouse line carries the pathogenic A30P mutation, which was introduced in the second exon of the mouse α-syn gene [44]. Although these mice express normal levels of A30P mutated α-syn, they show age-dependent motor decline, coinciding with decreased levels of DA within striatum and mesolimbic area. Additionally, these mice showed significant dysfunction of VMAT2 activity, which suggests the importance of α-syn in DA neurotransmission [44]. Recently, another transgenic mouse model was generated, based on the transfer of a bacterial artificial chromosome carrying the entire human α-syn locus [45]. The mice were backcrossed to a mouse α-syn null background. These mice express human α-syn at a level which is increased nearly two-fold compared to wild-type animals. They display deficits in neurotransmission that are rather specific to the DA system in the dorsal striatum, and that are independent of neuronal degeneration. Overall, these results demonstrate dose-dependent effects of human α-syn on DA neurotransmission, which may contribute to the symptoms observed in PD.

### 2.2. Alpha-Synuclein and the Mitochondria

Alpha-syn interacts with mitochondria via its *N*-terminal domain [43]. Furthermore, the first 32 amino acids of α-syn may contain a putative mitochondrial localization signal [43]. Even though α-syn has been detected on the outer mitochondrial membrane, it can be translocated to the inner membrane via the TOM40 transporter [43]. The role of the interaction between α-syn and mitochondria still remains poorly understood. What is known however is that association of α-syn with membranes is highly dependent on lipid composition [46]. Alpha-syn binds preferably to membranes with highly negative charge at their surface. Additionally, its interaction with the mitochondrial membrane is facilitated by the presence of cardiolipin [46], a diphosphatidylglycerol lipid that is considered to be a scaffold chaperone for the components of electron transport chain (ETC) [47]. Hence, it has been postulated that binding of α-syn to the mitochondrial membrane might bring stability to the ETC complex. Additionally, it has been shown that α-syn has antioxidant capacity that is completely dependent on its association with cellular membranes [14,48]. This mechanism is based upon cyclic oxidation and reduction of two methionine residues situated in the *N*-terminal compartment of the protein. Cyclic oxidation takes place in the membrane, when the protein is in close proximity to peroxidized lipids, whilst methionine residues are reduced in the cytosol by the methionine sulfoxide reductase [14]. Even though this mechanism is not exclusive to mitochondrial membranes, it is probable that mitochondria, due to their high metabolic activity, are the major target for α-syn antioxidant activity.

## 3. Pathological Role of α-Syn

The importance of α-syn in the PD pathology has been first recognized with the identification of α-syn mutation in familial early-onset PD cases with a dominant mode of inheritance [1]. This was followed by the discovery that this protein is the major component of the LBs observed in *post-mortem* brain tissue [8]. Up to now, five missense mutations have been identified in the α-syn coding sequence, *i.e.*, the well-established A53T, E46K, A30P mutations and the recently discovered H50Q and G51D [1,2,3,4,5]. Later, other cases of familial PD with confirmed *post-mortem* pathology were found to bear duplication [6] or triplication [7] of the α-syn gene. Disease onset and gravity of symptoms is clearly dependent on α-syn dose [6], and these patients also show non-motor symptoms [6]. Genetic variations in the first exon of the α-syn gene also correlate with PD pathology. More specifically, polymorphisms, variations in length or hypomethylation are found in the promoter region of α-syn, and are overall likely to affect the level of α-syn expression [32]. Finally, genome wide association studies (GWAS) identified over 800 single nucleotide polymorphisms (SNPs) in α-syn gene that have been linked to the sporadic occurrence of PD [32,49,50,51].

**Figure 1:**
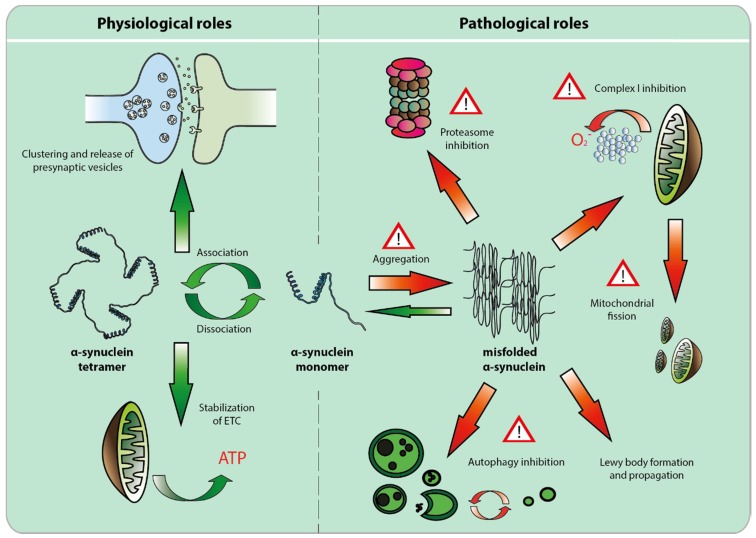
Physiological and pathological roles of α-synuclein. Alpha-synuclein, in physiological conditions, is considered to exist in two forms, monomer and tetramer. Its role in neuronal homeostasis is mostly associated with molecular chaperone activity at the synapse, where it controls clustering [39,40,41] and release of synaptic vesicles [33,34,42], and at the mitochondrial membrane, where together with cardiolipin, it stabilizes electron transport chain proteins [47]. When misfolded and/or aggregated, α-synuclein inhibits the ubiquitin-proteasome system [52,53,54,55] as well as mitochondrial complex I activity [43,56,57], blocks mitochondrial fusion [58,59] and impairs autophagy [60,61,62].

In conclusion, although genetic variants of α-syn represent only a fraction of PD cases, these findings have built a strong argument for the central role of this protein in PD pathogenesis. Since α-syn is the major component of LBs present in the vast majority of PD patients, it appears likely that it might play a key role in the mechanisms leading to PD, no matter what the genetic or environmental cause exactly is. The involvement of α-syn in physiological and PD-related processes is summarized in Figure 1.

### 3.1. Alpha-Syn Misfolding, Aggregation and Propagation

Lewy Bodies, the most typical pathological hallmark of PD brain, are aggregates mainly composed of high molecular weight α-syn fibrils. The nature of LBs and their role in PD pathology has not been completely established, and it is considered that LBs are either a toxic entity or a manifestation of cellular defense mechanisms against persevering pathology. Alpha-syn is present inside the cell in several forms, *i.e.*, as monomer, oligomer, protofibril or mature fibril. Interestingly LBs are mostly built up of mature fibrils, entities that are rather inert compared to their oligomeric counterparts [13].

Oligomers seem to be generated at the surface of cellular membranes, during the process of NAC fragment exposition and the formation of beta-sheets [13,63]. It is however unclear how the A30P mutant, which has a lower propensity to interact with cellular membranes, is in fact able to generate oligomeric species and show pathogenic effects. Of note is that the natural capacity of α-syn to aggregate or interact with cellular membranes can be modified via post-translational modifications like phosphorylation, nitration or oxidation [9,64,65,66,67].

Interestingly, α-syn pathology is not only confined to the DA neurons, but is also present throughout the nervous system, as well as peripheral tissues. In PD, synucleinopathy affects chronologically first the colonic nerves, adrenal medulla, the cardiac sympathetic system [68,69], and olfactory bulb. The pathology of these systems coincides with early premotor symptoms of PD, like constipation [70], cardiac dysautonomia [71], increased anxiety [72,73,74] and loss of olfaction [75,76], respectively. The adrenal medulla and cardiac sympathetic systems express catecholamines, and therefore share with nigral DA neurons some enzymes and precursors involved in catecholamine biosynthesis.

CNS structures seem to be affected in a sequential and progressive fashion, as described in the Braak staging of the α-syn pathology in PD [22]. It is hypothesized that PD pathology, as well as some other neurodegenerative diseases, might in fact have a similar way of development as “prion-like” diseases, with α-syn being the “infectious particle” [9,13,77]. The clinical evidence supporting this notion comes from the observation that PD patients grafted with fetal DA progenitors develop α-syn pathology within the transplanted neurons a few years later [78,79]. A series of experiments pointed out the possibility that α-syn may spread from one cell to another in the form of monomers or oligomers, secreted from the donor cell, or via exocytosis in vesicles [14,80]. Once in the acceptor cell, it may serve as a “seed” or matrix to induce misfolding of residential protein material, hence spreading pathology to a novel cell [13,81,82,83].

Conversely to what was initially assumed, LBs are not PD exclusive. In fact, α-syn deposits can be found in several neurodegenerative disorders such as dementia with Lewy Bodies (DLB) [8,84], Alzheimer’s disease (AD) [8,85] and multiple system atrophy (MSA) [86]. What distinguishes these aggregates from those found in PD is their different pattern of distribution within the brain. In DLB, they are mostly present in the cortex, for AD in the amygdala, whilst in the case of MSA they can be found in glial cells [9]. LBs can be found in asymptomatic aged individuals without any evident nigral degeneration [87]. This may suggest that LBs might naturally occur, along with normal aging [21,87]. This notion is however regarded as unlikely and these patients are generally considered to be in a presymptomatic stage of PD [21,87].

### 3.2. Loss of Function Toxicity

One proposed cause of α-syn toxicity might in fact be related to the loss of its physiological function [13]. This could be a result of post-translational modifications, pathogenic mutations, misfolding, or entrapment of the protein within LBs. No matter how it happens, the final result would be the loss of functional α-syn units where it normally exerts its physiological role.

Of key importance is the structural nature of this “functional unit” inside the DA neurons. Some controversy exists regarding α-syn structure in normal conditions. One theory suggests that α-syn is of a great majority in a monomeric state [13], whilst it is also proposed that α-syn is mostly in a tetrameric form [88,89]. Alpha-syn tetramers might in fact be more resistant to misfolding and aggregation than the monomers [88]. This notion is supported by data from *post-mortem* brain analysis, where mutated α-syn, with a higher propensity to aggregate, had significantly lower α-syn tetramer-to-monomer ratio [89]. This indicates that α-syn molecules might be in a dynamic equilibrium in the cytosol. Shifts towards the monomeric form might induce loss of the protein function, and trigger its misfolding and eventual toxicity.

Of note, even though α-syn null mice are viable and do not show any particular signs of impairment, a recent study by Manfredsson *et al.*, has shown that AAV-driven down-regulation of α-syn in rats and African Green monkeys, via intranigral delivery of shRNA against α-syn mRNA, leads to a significant degeneration of DA neurons [90,91]. The region most prominently affected by the degeneration was the ventral tier of the SNpc. This might indicate that the survival of adult DA neurons in the ventral midbrain requires α-syn to be expressed. Although highly interesting, the primate study needs to be reproduced since it involved only a small number of animals. Nevertheless, these results indicate the potential importance of α-syn for the survival of nigral DA neurons in the adult primate brain.

### 3.3. Alpha-syn Toxicity Affects Multiple Compartments, but Mitochondrial Toxicity May Prevail

Even though the loss of α-syn function may play a role in PD, a significant body of evidence indicates that α-syn gains toxic functions in pathological circumstances. Overall, α-syn exerts its multifaceted toxic effects in multiple cellular compartments affecting processes that are of major importance for neuronal homeostasis and functionality [13,30,92]. Apart from inducing unfolded protein response (UPR), *i.e.*, endoplasmic reticulum related stress [93,94,95], α-syn may also disrupt cytoskeleton architecture [96], impair proteasome’s function [52,53,54,55] and affect autophagy [60,61,62]. However, most significant toxicity triggered by α-syn seems to affect mitochondria.

Almost since the dawning of PD research, mitochondria were of major interest as a potential key to understanding the mechanisms of pathology. This was mostly because mitochondria are a major source of reactive oxygen species (ROS). A clinical indication for the selective vulnerability of nigral DA neurons to alterations of the mitochondrial function came from the case of accidental intoxication with MPTP, which caused acute parkinsonism in a small group of drug addicts [17]. MPTP, which is a byproduct of the synthesis of the opioid drug MPPP, is metabolized into MPP+. This compound is a potent inhibitor of the mitochondrial complex I. Histopathological analysis of the brains exposed to the toxin revealed severe lesions, particularly in the nigrostriatal system [17].

Additional evidence supporting the importance of mitochondria came from the identification of other genes implicated in familial PD. In particular, mutations responsible for autosomal recessive juvenile PD (AR-JP) have been found in the genes encoding Parkin, PINK1 and DJ-1. These proteins have crucial roles in mitochondrial homeostasis. PINK1 kinase and its downstream partner Parkin, an ubiquitin E3 ligase, work in concert, leading to the ubiquitination of mitochondrial components. This process induces the clearance of dysfunctional mitochondria [97,98,99]. In addition, Parkin has been implicated in the biogenesis of mitochondria by promoting the expression of peroxisome proliferator-activated receptor gamma coactivator 1-alpha (PGC-1α) via the proteasomal degradation of the PARIS repressor [100]. Finally, DJ-1 is a molecular chaperone, whose role is to protect the DA neurons from the advert effects of oxidative stress [101].

Mutations in the LRRK2 gene are associated to familial PD cases with dominant inheritance [102]. Although the physiological role of LRRK2 is not yet fully understood, it has been shown that LRRK2’s kinase activity mediates mitochondrial fission and fusion dynamics [102,103] and that the LRRK2 G2019S mutation renders mitochondria more sensitive to toxins [102,104].

Although no major pathway linking the pathological roles of all PD-associated genes could be identified yet, it appears that the mitochondria might represent a common denominator among the different causes of PD, as it is also clearly affected by α-syn. Indeed, accumulated α-syn (misfolded or overexpressed) is capable of inhibiting the activity of mitochondrial complex I [43,56,57]. This induces oxidative stress via increase in superoxide production. Experimental models show that α-syn contributes to the activity of PD-related toxins. Overexpression of a mutated form of α-syn increases rotenone-dependent toxicity [105], whilst α-syn knockout mice are resistant to MPTP intoxication [106]. It appears likely that α-syn mediates these toxic insults in part via its interaction with mitochondria.

It has further been shown that α-syn inhibits the fusion of mitochondria and to alter the structure of their cristae [58,59]. The resulting mitochondrial fragmentation might induce clearance of the organelles via autophagy, since smaller mitochondria are more likely to become targets for mitophagy [107]. Finally, α-syn impairs the transport and delivery of mitochondria to their destination via interaction with the cytoskeleton network and motor proteins [108,109]. This could have a significant impact on DA neurons that normally exhibit a high energetic demand. Furthermore, it would also explain why these long-projection neurons might be so sensitive to the α-syn pathology.

## 4. Aging as a Major Risk Factor for PD

Aging constitutes a major risk factor for PD. Some of the age-related changes in behavior closely resemble those observed in PD patients, such as slowness of motion, postural instability and cognitive deficits. Hence, PD is often considered as a form of accelerated aging. However, there are also a number of indications that point out to major divergences between normal aging and the pathology observed in PD. Strongest evidence comes from *post-mortem* brain analysis, which shows different patterns of neuronal loss [21]. In PD, the population of DA neurons that is most affected by the pathology is in the ventral tier of SNpc. Conversely, it is the dorsal part of the very same structure that seems to degenerate most preferentially in aging [21]. It is worth mentioning that these two populations differ at both biochemical and structural levels [21,23], with the dorsal tier being more similar to the DA neurons of the VTA [23].

**Figure 2:**
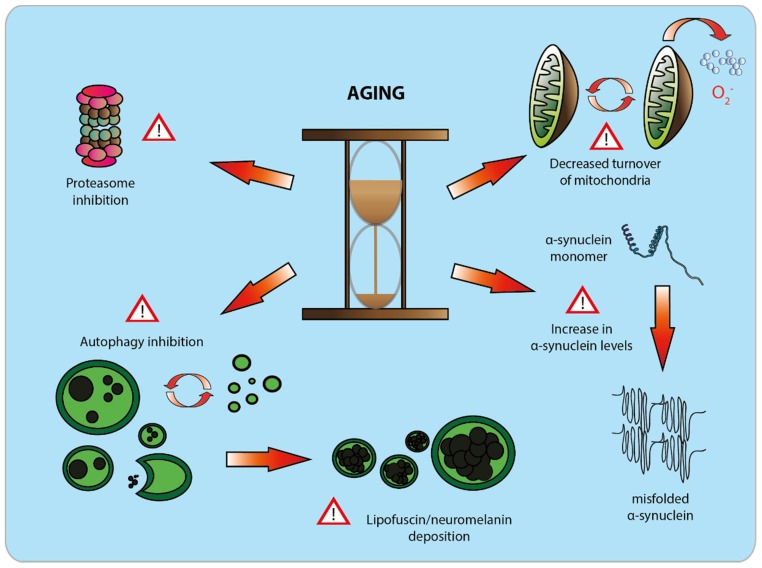
The effect of aging on dopamine neurons. During aging, dopamine neurons of the *substantia nigra pars compacta* undergo a series of debilitating molecular changes, which compromise their physiological function. With time, a decrease in mitochondrial turnover occurs. As a consequence of this, accumulation of dysfunctional mitochondria is observed, which results in an increase in reactive oxygen species production [29]. Ubiquitin-proteasome and autophagic activities gradually decline with time [27,28], which leads to the deposition of neuronal pigments like neuromelanin or lipofuscin [114]. Aging has been correlated to a significant increase in protein levels of α-synuclein, promoting misfolding and neuronal toxicity [111].

The aim of this section is to bring similarities and indicate differences between aging and PD, in order to better understand the nature of the interplay between these two processes. The information is summarized in Figure 2, which shows the mutual relationship between aging and PD, with a focus on DA neurons.

### 4.1. Structural Changes in the Aging and Diseased Brain

The brain is obviously a key structure in the human organism, which requires 20% of the total energy supply, whilst weighing only 2% of its total mass [110]. Neuronal cells highly depend on energy for effective neurotransmission. As we age, brain performance declines, both at the motor and cognitive levels. At the cellular level, this might be due in large part to metabolic changes in the brain. As a consequence, neurons may lose their functionality and plasticity and/or degenerate and die over time. Nevertheless, the brain as a system copes surprisingly well with the process of aging, despite the low rate of renewal of neurons throughout life.

Only some of the brain regions appear to be particularly vulnerable, whilst the majority of them do not degenerate and remain perfectly functional [20]. For example, hippocampus, putamen, hypothalamus or nucleus basalis of Meynert remain almost completely intact over time [20]. Other brain structures, like the neocortex, lose on average 10% of the total number of neurons throughout life [20]. On the other hand, some brain nuclei seem to be particularly sensitive to the aging process. Among these, ventral tegmental area (VTA), retrorubral area and the SN are some of the most severely affected [20]. Remarkably, all these regions mainly use DA for neurotransmission, which hints at a possible link between DA and higher sensitivity to age-related degeneration. Neuron loss starts in the SNpc typically around the age of 40, and was initially claimed to progress at the rate of 10% with every decade [20]. However, thanks to more advanced stereological methods for estimating neuron numbers, this rate has been adjusted to be around 4.7% per decade [20]. This is however a matter of controversy, since a recent series of analyses opts for TH marker loss, rather than DA neurons’ death [111,112]. This notion is appealing, considering that α-syn protein levels increase with age and that α-syn overexpression can affect the expression of DA markers [111]. According to a recent study based on MRI, degeneration of DA fibers, associated with a decline in DA neurotransmission, can be observed in normally aging individuals. Nevertheless, degeneration is substantially less evident than in PD patients [113]. Remarkably, the structures that are most sensitive to age-related atrophy are as well most severely degenerating in PD [113].

SNpc is most affected by PD and for many years, it has been considered as the focal point for the origin of PD symptoms [20,115]. It is assumed that an individual develops the first symptoms when 50% of the nigral DA neurons have degenerated [115]. In the striatum, the degeneration of the DA axonal fibers is even more extensive, with a concomitant 80% decrease in DA content [115]. It has been shown that the PD pathology is of a highly acute nature within the nigrostriatal system [21,116]. In fact, most of the neurons and fibers positive for TH and the dopamine transporter (DAT) are lost within the first 4–5 years after disease diagnosis [21,116], with a relatively slow progression observed afterwards. Interestingly, this TH and DAT decline does not depend on the age of an individual but rather on disease duration [21,116]. The loss of pigmented neurons in the SNpc does not follow the same kinetics as the DA marker loss [116]. This might suggest that one of the first events in PD pathology is the loss of the DA phenotype and decrease in neuronal activity. Neuron and fiber’s death may take more time, which hopefully leaves a window for potential therapeutic intervention after disease diagnosis.

### 4.2. Cellular Alterations in the Aging and Diseased Brain: Neuronal Pigments

One accurate indication of brain aging at the cellular level is the presence of intracellular pigments, *i.e.*, lipofuscin and neuromelanin [25,107,114]. These pigments, even though they appear approximately at the same time in the aging brain, possess different characteristics [114].

Lipofuscin is a yellow-brownish, autofluorescent pigment present virtually in all brain compartments. It is found in neurons and occasionally in glial cells [107,114]. Lipofuscin accumulation could be caused by autophagic activity, constituting a non-digestible lipid and protein material within the lumen of autolysosomes [25,107,114]. On the other hand, neuromelanin is a dark, non-autofluorescent pigment. Its production is not only specific to neurons, but also almost exclusively restricted to catecholaminergic neurons, mainly in the SNpc [114,117,118]. In some instances, however, it may be found in other parts of the brain [118], as well as in microglial cells [118]. The latter case, however, could be a direct consequence of the phagocytosis of neuronal debris by activated microglia, rather than active deposition of the pigment [118]. The reason why neuromelanin is synthesized mostly by catecholaminergic neurons still remains a matter of debate. Nevertheless, it is postulated that it is generated by autoxidation of free cytosolic DA [114,117,118]. Indeed, the deposition of neuromelanin in human DA neurons is significantly decreased where the vesicular monoamine transporter VMAT2 is highly abundant [119].

Both of these pigments do not seem to coexist at the same time in one neuron [114]. Although they might share some steps in their synthesis, their role in neurons is proposed to be different [114]. As such, lipofuscin is considered to be a molecular waste of cellular metabolism, which in some instances may even be toxic for the cell [25,107,114]. On the other hand, neuromelanin is considered to be mainly protective in catecholaminergic neurons. This is mainly due to its ability to chelate metals, mostly iron [114,117,118], the level of which increases gradually with age. Neuromelanin may also bind mitochondrial toxins such as MPP^+^ or paraquat. Finally, it eliminates cytosolic, non-vesicular DA, hence protecting the neuron from oxidative stress related to its autoxidation [114,117,118].

Regarding the importance of lipofuscin and neuromelanin in the process of aging, both of these pigments appear in the aging brain more or less at the same time, *i.e.*, at the age of 3–4 years [114] and they linearly accumulate over time [107,114]. In the case of PD, however, the number of neuromelanin positive neurons is drastically diminished compared to age-matched individuals. In contrast, neurons containing lipofuscin remain largely unaffected [114]. It has been shown that DA neurons have less neuromelanin in PD patients than in healthy individuals [117]. What is more, neurons that die off in PD seem to have less neuromelanin than those surviving [20,117].

The protective role of neuromelanin remains, however, controversial in the context of PD. Neuromelanin constitutes a reservoir of highly toxic and oxidative entities, which can be released once the pigment is overloaded, degraded or released into the extracellular milieu [114]. When iron is in excess, it becomes bound by low affinity sites on neuromelanin, which does not hinder its oxidative effects via the Fenton’s reaction. Additionally, components of neuromelanin, once released into the extracellular space, may induce a significant inflammatory response and microglial activation [114].

### 4.3. Molecular Changes in the Aging and Diseased Brain: Proteostasis

Another feature that aging and PD pathology share in common is the impaired protein homeostasis (proteostasis), due to the failure of the protein quality control systems [13,20,26,27,28,96]. As we age, the ubiquitin proteasome system (UPS) and autophagy, two major pathways responsible for degradation of misfolded or aggregated proteins, decline in efficacy. This is caused by the loss of activity of key components in both systems, e.g., via decreased renewal, protein oxidation, molecular chaperones’ dysfunction [27,28] or indirectly via a decline in mitochondrial activity. The latter would eventually result in decreased production of ATP, which is critical for UPS activity [26]. As a consequence, misfolded proteins may neither be cleared from the system nor folded back into the correct structure [26]. This may cause further protein misfolding (template effect) [120,121], leading to protein aggregation and ultimately cell death.

In PD patients, we can observe a significant drop in proteasomal activity [122], as well as impairments in autophagy [61,123,124,125]. According to *in vitro* studies, α-syn inhibits the activity of the proteasome [52,53,54,55], most probably via the recruitment of proteasomal subunits for the degradation of proteins accumulating in LBs. Indeed, it has been shown that proteasomal subunits can be physical components of LBs [53,126].

Regarding the effect of α-syn on autophagic activity, some initial reports showed that α-syn overexpression decreases autophagosome formation via defective ATG9 recruitment [62]. In accord with this, genetic and biochemical data indicate that components of chaperone-mediated autophagy (CMA) might be impaired in patients with an idiopathic form of PD [61,123,124,125]. This is particularly important since CMA, along with UPS, are considered major degradation pathways for monomeric α-syn [60,127]. PD is also characterized by the cytosolic retention of TFEB, a factor implicated in the transcriptional control of autophagy [128]. Remarkably, overexpression of TFEB protects nigral DA neurons from α-syn toxicity. However, some *in vitro* data obtained in neuronal cultures and yeast model of aging also indicate that wild-type as well as mutated α-syn can up-regulate macroautophagy and, as a consequence, induce clearance of mitochondria [129,130]. This would suggest that once the cell is exposed to high levels of α-syn promoting self-aggregation, neurons might activate macroautophagy as a compensatory mechanism aimed to reduce the misfolded protein burden [127]. However, as a result, collateral damage in the form of clearance of both functional and dysfunctional mitochondria might take place, which could lead to α-syn-induced DA neurons death in aged individuals [130].

Bearing in mind the key role that α-syn plays in PD pathology, it is worth mentioning the relationship between aging and α-syn itself. A clear correlation between aging and α-syn has been noticed in the plasma, where α-syn level declines with age [131]. Furthermore, in both monkeys and humans, elevated levels of the α-syn protein have been detected in the aging SNpc [111]. For humans, this increase reached up to 600% as compared to young individuals [111]. Interestingly, this phenomenon is α-syn-specific, since the level of another member of synuclein family, beta-synuclein, remains stable during lifespan [111]. Since mRNA levels for α-syn do not vary significantly between young and aged individuals, the observed differences are attributed to the limited capability of the cell to degrade accumulating α-syn [111], which would be perfectly in line with the aforementioned declines in proteasome and autophagy activities.

Unexpectedly, conclusions made for humans and non-human primates are in contradiction to what is observed in the mouse species, where both mRNA and protein levels of α-syn decline gradually with age [132]. The aging mouse brain counteracts, however, this drop by increasing α-syn post-translational modifications [133]. This phenomenon is in accord with the fact that oxidized forms of α-syn, similar to the A53T mutation, have longer half-lives and tend to accumulate in the aging mouse brain [133].

Alterations of the α-syn levels in aging human and primate brains are clearly region-specific. The most significant increase is observed within the SNpc [111]. In nigral DA neurons, the accumulation of the protein is most evident in the perikarya. Additionally, α-syn in this form is proteinase K sensitive, meaning that aging does not necessarily lead to irreversible fibrillization [111]. Of note, the accumulation of human α-syn in the rat SNpc appears to be decreased by the activity of FOXO3, a gene which has been associated with healthy aging in humans [134].

### 4.4. Changes in Mitochondrial Activity

Another cellular signature of aging is the alterations in mitochondrial activity. It manifests itself with a progressive decrease in efficacy of beta-oxidation, oxidative phosphorylation and Krebs cycle [29]. The reason for this is most probably because oxidative phosphorylation occurs in mitochondria, exposing this organelle to the adverse effects of oxidative stress. This may result in cumulative damage, which progressively impairs their performance [29].

One of the most vulnerable parts of mitochondria is their genetic material, existing in the form of multi-copy double stranded circular DNA [29,135,136,137]. Due to the lack of histones and effective proofreading systems [29], mitochondrial DNA (mtDNA) is a relatively easy target for ROS, which may result in point mutations and deletions. Such deletions need time to accumulate or clonally expand in the cellular pool of mitochondria. Indeed, due to lack of reparatory mechanisms, the abundance of these alterations correlates in a linear fashion with the age of the cell [29,135].

Yet again, similar changes in mitochondrial biology and activity do occur in PD as well. The most frequently observed alterations are decreased activity of complex I, complex IV, accumulation of mtDNA deletions, as well as the presence of swollen and dysfunctional mitochondria inside neurons [135,138].

Molecular evidence for the decline of mitochondrial activity also comes from the meta-analysis of gene expression in *post-mortem* tissue from the SNpc of PD patients. A clear correlation is indeed found between the occurrence of PD and a decrease in the activity of PGC-1α [139]. PGC-1α is a transcriptional co-activator, which is a master regulator of the expression of nuclear genes implicated in mitochondrial biogenesis, as well as oxidative phosphorylation [139,140]. PGC-1α also induces the expression of genes implicated in the cellular resistance to oxidative stress [141]. The relevance of PGC-1α in PD has been reinforced with additional clinical observations that diabetic patients treated with glitazone, a PPARγ receptor agonist, show decreased morbidity of PD [142]. The transcriptional repression of PGC-1α by PARIS induces degeneration of DA neurons in mice [100], an effect that is reversed when PGC-1α expression is rescued [100]. Additionally, PGC-1α deficiency accelerates the formation of α-syn oligomers, which is rescued via treatment with AICAR or resveratrol [143]. Conversely, α-syn has been shown as well to be able to directly interact with the PGC-1α promoter and inhibit its transcription [144]. Finally, the nigral DA neurons of PGC-1α null mice, which accumulate defective mitochondria, are more vulnerable to α-syn toxicity [140]. These reports indicate that neurons with low PGC-1α activity enter a vicious circle, where interaction between PGC-1α and α-syn plays a major role. The aging process may further catalyze this detrimental process.

## 5. Dopaminergic Neurons of SNpc—Colossus with Feet Made of Clay

Even though there seems to be a difference in the pattern of cell death within SNpc between normal aging and PD [21], DA neurons appear to be selectively vulnerable to both of these phenomena. Therefore, it is important to discuss the characteristics that render them more susceptible than other types of neurons.

Many theories have been proposed to explain why these neurons are so sensitive. One can cite age-dependent accumulation of iron [145,146], increased oxidative stress due to DA metabolism [147], or neuromelanin degradation [114]. All these factors are likely to collectively contribute to the pathogenesis, possibly via increased vulnerability to α-syn accumulation. However, DA neurons in the SNpc also appear to have a complex architecture, which may pose specific problems, including the management of energy supply [23,148,149].

One of the peculiarities of PD is that it affects mostly neurons that possess long, unmyelinated axons, like DA neurons in the SNpc, or cholinergic neurons in the peduculopontine nucleus and nucleus basalis of Meynert [23,149]. Since unmyelinated axons generally require more energy in order to propagate action potentials, one might conclude that neurons with higher energetic demand to maintain their activity are more susceptible to degenerate.

Another argument, which seems to further support the idea that cells that are energetically compromised would be more vulnerable to pathology, comes from the complexity of the axonal structure. A single DA neuron situated in the ventral tier of the rat SNpc innervates approximately 6% of the total volume of the striatum [23]. Additionally, with around 80 cm total axonal length [23,148], each neuron has over 200,000 synaptic connections, exclusively with medium spiny neurons [23]. For comparison, the rat medium spiny neurons present in the striatum have in total only about 300–500 synapses per neuron [23]. Therefore, rodent DA neurons have an impressive network of synaptic connectivity, and as a consequence, very high ATP demand for propagating action potential and for recovering ion gradients across the membrane [23,149]. However, PD does not naturally occur in rodent species, which might indicate that axonal architecture is an important, yet insufficient component for acquiring PD. Most recent computational models take into account the fact that human striatal volume, as compared to rat, has increased over 300-fold as a consequence of evolution, but at the same time, the number of DA neurons within SNpc has increased only 32-fold. The unequal evolutionary “growth” of these two structures, renders the human DA system over 10 times more overwhelmed than that of its rat counterpart [23]. This might explain why humans, as compared to rats, are more prone to disease. Therefore, as we get older, the system might no longer be capable of fine-tuning the balance between energy supply and its efficient utilization, therefore causing more and more cumulative damage to the neurons.

Neurons require significant energy supplies to upkeep ion gradients across the membrane and to efficiently propagate action potentials. However, DA neurons of SNpc possess a series of features that seem not to be adequate for their high-energy demand. Firstly, pacemaker activity of nigral neurons, which grants the constant volume transmission of DA, is highly dependent on very energy costly L-type calcium channels [149]. Secondly, nigral DA neurons contain smaller mitochondria than those of other neurons [150,151]. In addition, the mitochondrial mass of mouse nigral DA neurons occupies a significantly smaller fraction of the cytosol than in their non-DA counterparts [151]. Finally, because of the massive axonal arborization, the transport of mitochondria along the cytoskeletal tracts and their delivery to high-energy demanding cell compartments may be challenging for DA neurons. Indeed, *in vitro* studies indicate that not only the cytoskeleton is highly sensitive to insults related to PD, such as MPP+ intoxication, but also that the transport of mitochondria within DA axons is slower than in other neuronal cell types [23,150]. Furthermore, α-syn has been shown to interact with motor proteins like kinesin, impairing axonal transport [152] and hence affecting the distribution of mitochondria along the neuron.

All in all, a proper balance between energy demand and its supply is key for the fine-tuned action of every neuron in our brain. DA neurons present in the SNpc have developed an extreme morphology, which may impose significant metabolic stress to these cells. Exposure of these neurons to the progressive deleterious effects of aging may cause their selective vulnerability to pathogenic insults. Therefore, it appears important to experimentally explore how this process interacts with factors that play an important etiologic role in PD, such as α-syn accumulation.

## 6. Animal Models of Aging

If one wishes to investigate in greater detail the interplay between aging and PD, one needs a solid animal model that recapitulates at least some of the aforementioned molecular aspects of aging. Due to natural time limitations related to modeling of aging in animals, most of the models utilize genetic tools to speed up the kinetics of the process. In line with what was mentioned before, mitochondria is one of the most promising targets to induce age-related changes in an accelerated fashion. There are several ways of introducing either mitochondrial mutations or deletions in mtDNA to trigger age-related phenotypes.

One way to accomplish this is to introduce genetic alterations in the proofreading system responsible for mtDNA replication. This was achieved with the creation of the so-called “Mutator mice”, animals that carry mutations in both copies of their nuclear encoded mtDNA polymerase (POLG), in order to alter the 3'–5' proofreading activity [153]. Homozygous animals obtained using this approach are characterized by a substantial amount of random mtDNA point mutations (20–30 mutations per mtDNA molecule), as well as 10–100 times more mtDNA deletions than their wild-type littermates [153,154]. All of these mutations introduced to mtDNA result in severe fertility problems, early onset of osteoporosis, kyphosis, substantial hair loss and heart hypertrophy [153]. At the level of mitochondrial activity, a decrease in respiratory function was observed, which is attributed to increased degradation of proteins implicated in oxidative phosphorylation [153,155].

A significant degeneration of nigral DA neurons as a consequence of mtDNA mutations or deletions would possibly link PD to the mitochondrial defects associated with aging. However, this has not been established, probably because of the relative inert nature of the point mutations, or an insufficient number of mtDNA deletions. Indeed, in patients that carry POLG mutations, only high numbers of mtDNA deletions can trigger a significant neuronal death within the SNpc [154]. Hence, other mouse models are based on even more severe alterations within the structure of mtDNA, like double-stranded nicks (mito-PstI model) [156] or decrease in total amount of mtDNA (Twinkle model) [157].

One of the most spectacular examples of these strategies is the MitoPark mouse, with a cell-specific knockout (DAT promoter driven expression of Cre recombinase) of the mitochondrial transcription factor A (TFAM) [20,158]. TFAM is a key transcription factor for mtDNA, which not only controls the copy number and maintenance of mtDNA in the mitochondrial pool, but is also required to initiate transcription from mitochondrial promoters [158]. TFAM knockout animals accumulate with age a significant amount of mtDNA mutations within midbrain DA neurons. Furthermore, these mice show progressive motor impairment, already at the age of 14–15 weeks, which are reversible with l-DOPA administration. Interestingly, the beneficial response diminishes with time, which indicates a close resemblance with the human form of PD [158]. Additionally, the progression of motor impairment clearly correlates with the loss of the TH marker, both in the striatum and in the SNpc, with the VTA being less affected [158]. Finally, already at the age of 6 weeks, despite having no reported DA neuron loss, these animals have clear intracellular deposits that are negative for α-syn. These inclusions are most probably clusters of degenerating mitochondria, as they were still present even when these animals were backcrossed with α-syn null mice [158]. At the same time, even though DA neurons in this model experience a severe crisis in the mitochondrial function, no obvious increase in ROS production was observed, which suggests that impaired mitochondrial function does not necessarily lead to ROS elevation.

Genetically modified mice described in this section are among the first examples of models which may be used to explore the effect of aging on PD pathology. With recent improvements in our understanding of the molecular mechanisms underlying the aging process, an increasing number of transgenic animals will be available in the near future to explore the consequences of aging. It will be important to determine how the aging process affects the level of α-syn accumulation, or increases neuronal susceptibility to the toxic effects of this protein.

## 7. Conclusions

Ninety percent of all diagnosed PD cases have a multifactorial origin, which is likely to combine genetic and environmental components. Changes in the expression level and folding state of the α-syn protein, combined with the formation of various α-syn multimeric species, define the transition towards pathological conditions. Although it is recognized that aging is a major risk factor for PD, the time-dependent molecular changes that underlie the development of the pathology are only partially understood. Rationally, pathways implicated in protein and organelle recycling by the proteasome and autophagy, as well as the biogenesis and quality control of mitochondria are gaining attention, because they are critically affected both in PD and aging. In neurons exposed to the combined effects of α-syn and aging, these cellular mechanisms may undergo vicious circles precipitating neuronal demise. However, compared to normal aging, PD pathology has clear specificities showing that this process cannot be merely considered as a form of accelerated aging. Therefore, it is critical to explore the effect of the α-syn pathology in the context of the neurons that are selectively vulnerable to the converging effects of aging and α-syn proteotoxicity. In particular, the morphology and the metabolic needs of DA neurons, together with molecular specificities associated with DA neurotransmission, are critical factors for α-syn to exert its toxic effects. Using animal models dedicated to the study of the aging process, it will be important to understand the interaction between aging and α-syn in nigral DA neurons. By identifying therapeutic targets in this context, disease-modifying treatments may be found, that could be applicable to a broad population of patients.
